# Broad-Spectrum Antifungal, Biosurfactants and Bioemulsifier Activity of *Bacillus subtilis* subsp. *spizizenii*—A Potential Biocontrol and Bioremediation Agent in Agriculture

**DOI:** 10.3390/plants12061374

**Published:** 2023-03-20

**Authors:** Karina Guillén-Navarro, Tomás López-Gutiérrez, Verónica García-Fajardo, Sergio Gómez-Cornelio, Eugenia Zarza, Susana De la Rosa-García, Manuel Chan-Bacab

**Affiliations:** 1 Grupo Académico de Biotecnología Ambiental, Departamento de Ciencias de la Sustentabilidad, El Colegio de la Frontera Sur Unidad Tapachula, Carretera Antiguo Aeropuerto km 2.5, Tapachula 30700, Chiapas, Mexico; kguillen@ecosur.mx (K.G.-N.); eugenia.zarza@ecosur.mx (E.Z.); 2 Facultad de Ciencias Biologicas, Universidad Autónoma de Campeche, Av. Agustín Melgar s/n, Col. Buenavista, Campeche 24030, Campeche, Mexico; 3 Ingeniería en Biotecnología, Universidad Politécnica del Centro, Carretera Federal Villahermosa-Teapa km 22.5, Villahermosa 86290, Tabasco, Mexico; sagomezcornelio@gmail.com; 4 Laboratorio de Nanotecnología-CICTAT, División Académica de Ingeniería y Arquitectura, Universidad Juárez Autónoma de Tabasco, Carr. Cunduacán-Jalpa de Méndez km 1, Cunduacán 86690, Tabasco, Mexico; 5 Investigadora CONACyT—El Colegio de la Frontera Sur. Av. Insurgentes Sur 1582, Col. Crédito Constructor, Benito Juárez, Mexico City 03940, Mexico City, Mexico; 6 Laboratorio de Microbiología Aplicada, División Académica de Ciencias Biológicas, Universidad Juárez Autónoma de Tabasco, Carretera Villahermosa-Cárdenas km 0.5, Villahermosa 86000, Tabasco, Mexico; 7 Departamento de Microbiología Ambiental y Biotecnología, Universidad Autónoma de Campeche, Av. Agustín Melgar s/n, Col. Buenavista, Campeche 24030, Campeche, Mexico

**Keywords:** bioemulsifier, biosurfactants, antifungal activity, lipopeptide

## Abstract

In this study, the antifungal, biosurfactant and bioemulsifying activity of the lipopeptides produced by the marine bacterium *Bacillus subtilis* subsp. *spizizenii* MC6B-22 is presented. The kinetics showed that at 84 h, the highest yield of lipopeptides (556 mg/mL) with antifungal, biosurfactant, bioemulsifying and hemolytic activity was detected, finding a relationship with the sporulation of the bacteria. Based on the hemolytic activity, bio-guided purification methods were used to obtain the lipopeptide. By TLC, HPLC and MALDI-TOF, the mycosubtilin was identified as the main lipopeptide, and it was further confirmed by NRPS gene clusters prediction based on the strain’s genome sequence, in addition to other genes related to antimicrobial activity. The lipopeptide showed a broad-spectrum activity against ten phytopathogens of tropical crops at a minimum inhibitory concentration of 400 to 25 μg/mL and with a fungicidal mode of action. In addition, it exhibited that biosurfactant and bioemulsifying activities remain stable over a wide range of salinity and pH and it can emulsify different hydrophobic substrates. These results demonstrate the potential of the MC6B-22 strain as a biocontrol agent for agriculture and its application in bioremediation and other biotechnological fields.

## 1. Introduction

Tropical fruits are economically important worldwide; however, they are susceptible to pathogen attacks that affect production by weakening the plant or fruit quality before and after harvest, reducing shelf life and causing yield losses of greater than 50% [1,2,3,4]. Fungal species of the phyla Ascomycota, such as the genera *Botryosphaeria*, *Ceratocystis*, *Fusarium*, *Glomerella* and *Colletotrichum* and Basidiomycota, e.g., *Armillaria*, *Erythricium*, *Ganoderma* and *Rigidoporus*, are phytopathogens numerous and significant [3]. For example, anthracnose is a fungal disease caused by *Colletotrichum gloeosporioides* Penz and Sacc, which provoke large yield and quality losses of up to 40 and 100% [5]. Current control strategies for various phytopathogenic fungi include synthetic fungicides. However, toxicological concerns and the development of pathogen resistance have accelerated the search for alternatives for disease control [6].

Chemical compounds used in agriculture and industrial waste implicated in their elaboration significantly alter the environment and trophic chains [7]. An alternative for the remediation of these sites impacted is to use surface-active compounds (surfactants) [8]. The problem is that surfactants currently used represent a momentary and partial solution since they are petroleum-derived chemical compounds that cause adverse effects on the environment due to their high persistence and difficulty to be degraded [9,10]. Despite this, chemical surfactants are mostly commercialized for low-cost production [11]. By 2021, global demand for surfactants amounted to approximately USD 41.22 billion, with an annual growth of 4.9%. It is expected to reach USD 57.8 billion by 2028 [12]. If this trend continues, surfactants will have a greater environmental impact than can be mitigated. Therefore, a global shift to environmentally friendly compounds with the same functions, such as biosurfactants (BS) and bioemulsifiers (BE), is needed.

Both BS and BE are compounds resulting from microbial metabolism and their molecular weight determines their function. BS are characterized by low molecular weight, reducing the surface tension between two phases [13]. Due to this characteristic, microorganisms that produce BS can grow on various water-immiscible substrates. BS are structurally and functionally diverse and display properties as detergency. They also play an important role in hydrophobic compounds’ dispersion, foaming, wetting and solubilization of hydrophobic compounds, specificity in high temperatures, pH and salinity [11,14,15]. They are highly selective, and have low toxicity and high biodegradability, making them compatible with the environment. Moreover, they are considered antimicrobial, antiviral and antifungal agents. In contrast, BE have a high molecular weight and can stabilize emulsions for extended periods but are less effective in reducing surface tension [16,17]. Multiple microorganisms, such as fungi and bacteria, can produce these compounds and have been studied for a long time. Among the most reported genera in the literature are *Candida*, *Acinetobacter*, *Pseudomonas* and *Bacillus* [18,19,20].

The genus *Bacillus* shows remarkable plasticity in synthesizing antibiotic compounds and is considered a natural factory of biologically active compounds such as lipopeptides [11]. Most *Bacillus* species can produce several lipopeptides, including iturins, fengycins and surfactins [21]. *Bacillus* lipopeptides can be cyclic or linear and consist of 7 to 11 amino-acid residues linked to β-amino or β-hydroxy fatty acids [21]. Cyclic lipopeptides have received attention in biocontrol by their potent antimicrobial activity, low toxicity and high biodegradability compared to their chemical counterpart, making them environmentally friendly candidates for biocontrol strategies [22]. The iturins, especially mycosubtilin, show strong antifungal activity [23]. Most act as biosurfactants due to their amphiphilic nature and have properties as viscosity reducers, hydrocarbon solubilizing and mobilizing agents and metal chelators for environmental and bioremediation applications [24].

Most antimicrobial agents are produced from organisms isolated from terrestrial sources. Recently, marine microorganisms have been investigated for novel natural products, including antibiotics; however, antifungal activity is scarcely studied [25]. In previous work, different *Bacillus* strains isolated from marine biofilms showed in vitro antifungal activity against *C. gloeosporioides*, *C*. *fragariae* and *Fusarium oxysporum* [26]. To delve into the identity, biological activity, chemical nature and detection of genes involved in the biosynthesis of the biosurfactant with antifungal activity, we performed biochemical test and genome sequencing of a *B. subtilis* (MC6B-22) isolated from a marine biofilm. Specifically, we determined the time of highest for bio-guided purification. Antifungal activity against various tropical fruit pathogens was tested to demonstrate its potential broad-spectrum capability. We also evaluated biosurfactant stability and bioemulsifier activity at extreme conditions of temperatures, pH and salinity.

## 2. Results

### 2.1. Morphological, Physiological and Biochemical Characterization and Molecular Identification of Bacillus subtilis MC6B-22 Strain

*Bacillus subtilis* MC6B-22 is a rod-shaped Gram-positive motile forming endospores. This strain forms oval spores positioned centrally. The morphological, physiological and biochemical characteristics of this *Bacillus* are given in Table 1. According to Bergey’s manual of determinative bacteriology [27], the strain MC6B-22 was closely related to the *Bacillus* group.

**Figure 1:**
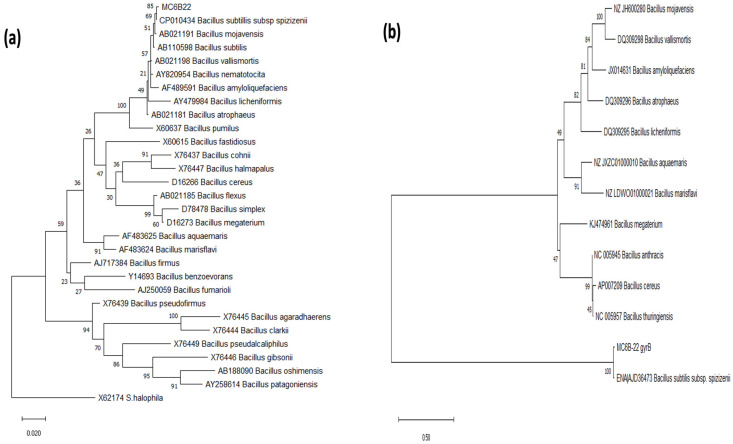
Maximum Likelihood Phylogenetic trees. Diagrams were obtained using 16S rRNA (**a**) and (**b**) Gyrase B Subunit gene sequences. The bootstrap consensus trees inferred from 1000 replicates were taken to represent the evolutionary history of the taxa analyzed. Branches corresponding to partitions reproduced in less than 50% bootstrap replicates are collapsed; the percentage of replicate trees is shown next to the branches.

Evolutionary relationship reconstruction using the phylogenetic markers (16s rDNA and *gyr*B) suggests that MC6B-22 has a closer phylogenetic relationship with *B. subtilis* subsp. *spizizenii* (Figure 1) than with other *Bacillus* species, including *B. mojavensis*.

### 2.2. Detection of Lipopeptides by PCR

Lipopeptides gene PCR assays indicated positive results using primers directed to surfactin, fengycin and mycosubtilin genes with a clear band of the expected size observed in gel (Table S1). The amplicon obtained using the iturin primers was shorter than expected (only 0.5 Kb). No PCR band was appreciated when using primers directed to bacillomycin D, although different PCR conditions were tested. With plipastatin primers, multiple bands were observed and were subsequently cloned for sequencing.

The sequences of PCR amplicons confirmed the surfactin and mycosubtilin identities, with similarity to surfactin synthetase *srf*AA (96% id), mycosubtilin synthetase *myc*B (93% id) from *B. subtilis* subsp. *spizizenii* as the best matches. With kurstakin primers amplicon, a region of 307 pb was similar to *srf*AA (93% id). The identity of amplicons from primers used for fengycin, plipastatin and iturin were similar to NRPS adenylation domain (99% id), methyltransferase *yod*H (99% id) and pullulanase *pul*A (100% id), respectively, from *B. subtilis* subsp. *spizizenii* strains. 

### 2.3. Genome Sequencing and Analysis

Genome assembly resulted in a 4,045,620 bp single contig, with 43.9% GC content. One hundred and forty-eight complete and single-copy BUSCOs were detected, suggesting we obtained a complete genome assembly. Results from the RAST database indicate that the MC6B-22 genome comprises 336 subsystems, 4169 coding sequences and 116 RNAs. Ten copies of the 16S rDNA were detected. BLAST searches were executed for each of them to confirm their identity. Three *Bacillus subtilis* strains appeared consistently as the top three hits of the BLAST searches, confirming that MC6B-22 belongs to the *B. subtilis* group, with a 100% identity with *Bacillus subtilis* strain FDAARGOS_606, 99.94–100% identity with *Bacillus subtilis* subsp. *spizizenii* str. W23 and *Bacillus intestinalis* strain T30 (Table 2). 

antiSMASH web server detected 11 regions of secondary metabolite biosynthesis gene clusters in the MC6B-22 genome (Table 3). Seven of them showed 100% of their genes matching known clusters. Two regions involved in terpenes synthesis were detected with other gene clusters with surfactant, antibiotic and antifungal activity. In this analysis, we confirmed detection of mycosubtilin activity in region 2 (Table 3). The identification of the mycosubtilin gene cluster with antiSMASH supports this. All genes in the cluster show similarity with mycosubtilin biosynthetic gene cluster from *B. subtilis* subsp. *spizizenii* ATCC 6633 (BGC0001103), followed by 66% similarity with the fengycine cluster (BGC0001095: fengycin biosynthetic gene cluster from *B. velezensis* FZB42) and 61% with the plipastatin cluster (BGC0000407: plipastatin biosynthetic gene cluster from *Bacillus subtilis* subsp. *subtilis*); 44% of the genes from this region show similarity with iturin (BGC0001098: iturin biosynthetic gene cluster from *B. subtilis*) (Figure S1).

The genomic analysis confirms the presence of the surfactin gene cluster in region 10 (Table 3), with 86% of genes showing similarity to the surfactin biosynthetic gene cluster from *Bacillus velezensis* FZB42 (BGC0000433) and less genes showing similarity to the lichenysin biosynthetic gene cluster from *Bacillus licheniformis* DSM 13 = ATCC 14580 (BGC0000381, 50%) and the basiliskamide A biosynthetic gene cluster from *Brevibacillus laterosporus* PE36 (BGC0000172, 9%). Additionally, we detected NRPS clusters for bacillaene (100%) (region 1), bacillibactin (region 5) and gene clusters related to antimicrobial metabolite syntheses like terpenes and rhizocticin A.

### 2.4. Growth Kinetic and Production of Crude Lipopeptide from Bacillus subtilis MC6B-22 with Antifungal, Biosurfactant and Bioemulsifier Activities

**Figure 2:**
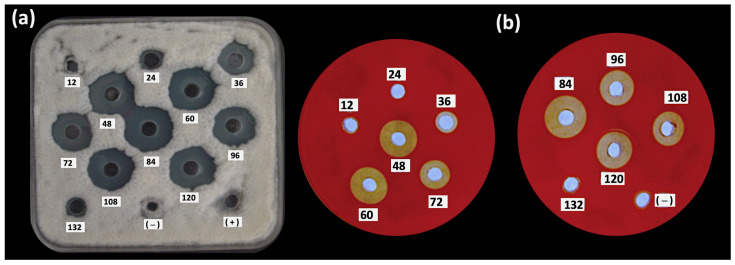
Kinetic of (**a**) antifungal (against *C. gloeosporioides* ATCC 42374) and (**b**) hemolytic activities over a period 132 h in LBMs of *B. subtilis* subsp. *spizizenii* MC6B-22.

In order to study the antifungal, biosurfactant and bioemulsifier properties produced during the growth of *B*. *subtilis* MC6B-22, the crude lipopeptide (CL) activity was evaluated at different time intervals (Figure 2). The activity produced by *B. subtilis* MC6B-22 was detected by bioassays from 24 h of culture (Table 4). The bioemulsifying activity continued to increase during the stationary phase until reaching a first peak of maximum activity at 84 h; after that, the activity declined at 96 h [39]. The production of antifungal and hemolytic compounds was evident from 36 to 120 h, finding the highest activity at 84 h, corresponding to the beginning of the death phase (Figure 2). At 84 h, the highest number of endospores was also found. In contrast, the biosurfactant activity increased in the exponential phase of 48 h and remained at 5–6 mm values until 96 h, which is the end of the stationary phase. The pH values increased to 8.49 during cultivation and the best CL yield was 556 mg/mL with the highest biosurfactant, bioemulsifying and antifungal activity against *C. gloeosporioides* ATCC 42374.

### 2.5. Partial Purification of Crude Lipopeptide Fraction

**Figure 3:**
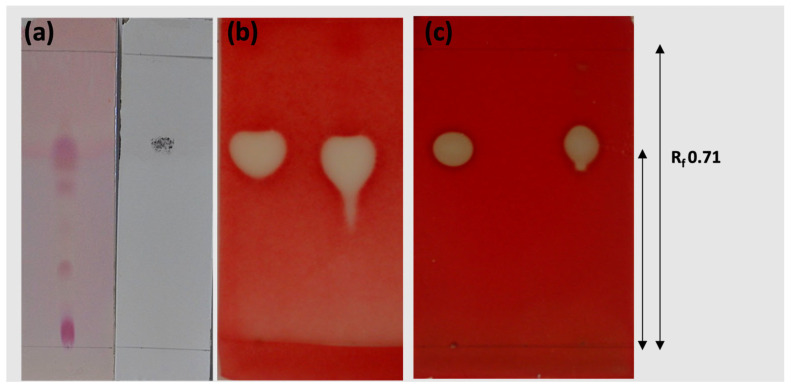
Retention factor in thin layer chromatography (TLC) analysis of the crude lipopeptide extract CL_84_. (**a**) revealed with ninhydrin and cupric sulfate. (**b**) Bioautography against *C. gloeosporioides* growth in an agar overlay and dyed with an aqueous solution of 2,3,5 triphenyltetrazolium chloride and (**c**) in blood agar overlay.

The crude extract of the lipopeptides at 84 h (CL_84_) with biological activity was prepurified by bioautography in TLC. The chemical identity of this bioactive biosurfactant fraction was determined with TLC plates revealed with cupric sulfate and ninhydrin, showing an R_f_ of 0.71 (Figure 3a) that coincides with the iturin and mycosubtilin standards. The bioautography assay shows in that same retention factor (R_f_) a halo of inhibition of mycelial growth of *C. gloeosporioides* (Figure 3b) and a zone of hemolysis (Figure 3c).

**Figure 4:**
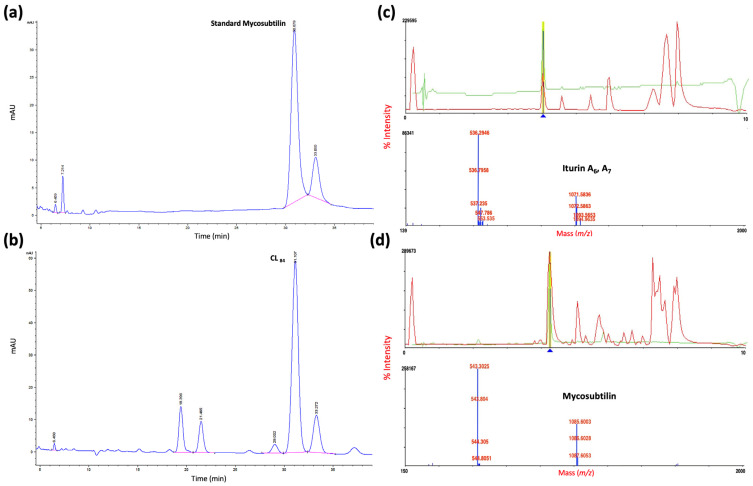
Characterization of lipopeptide for (**a**) HPLC standard mycosubtilin, (**b**) 84 h purified lipopeptide analysis (PL_84_); MALDI–TOF-MS analysis of (**c**) iturin A_6,_ A_7_ and (**d**) mycosubtilin.

The active area was scraped for characterization by a reverse-phase HPLC system. The active fraction PL_84_ coincided with the standard of mycosubtilin (Figure 4a,b) and by MALDI-TOF mass spectrometry detected iturin A and mycosubtilin (Figure 4c,d), with *m/z* values of 1071.5836 (iturin A_7_), [40,41] and 1085.6003 (mycosubtilin) [42] identified to form anteiso-17 mycosubtilin reported by [43] as novel mycosubtilin isoforms.

### 2.6. Antifungal and Bioemulsifier Activities

**Figure 5:**
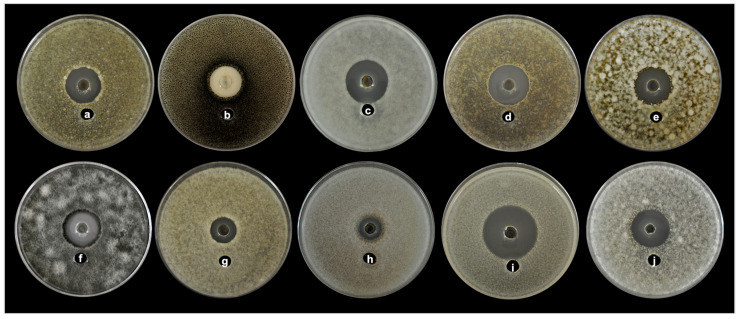
Antifungal activity of CL_84_ by agar well diffusion showing broad spectrum against different phytopathogens (**a**) *Aschochyta* sp., (**b**) *Colletorichum acutatum*, (**c**) *Colletotrichum gloeosporioide*s ATCC 42374, (**d**) *Colletotrichum gloeosporioides*, (**e**) *Colletotrichum capsici*, (**f**) *Curvularia clavata*, (**g**) *Fusarium nivale*, (**h**) *Fusarium solani*, (**i**) *Moniliophthora roreri*, (**j**) *Pestalotiopsis maculans*.

In the agar diffusion assay experiment, the CL_84_ extract showed an inhibition halo from 15.5 to 34 mm diameter of inhibition against all fungal plant pathogens (Table 5 and Figure 5). The CL_84_ proved to be a broad-spectrum antifungal, where *Moniliopthtora roreri* was most effectively inhibited (34 mm). At the same time, the strains of *Fusarium* were more resistant. The minimum inhibitory concentrations (MIC) values were from 12.5 to 100 μg/mL (Table 5). The mechanism of action against all phytopathogenic fungi evaluated was fungicidal; only for *Pestalotiopsis maculans*, the effect was fungistatic. 

**Figure 6:**
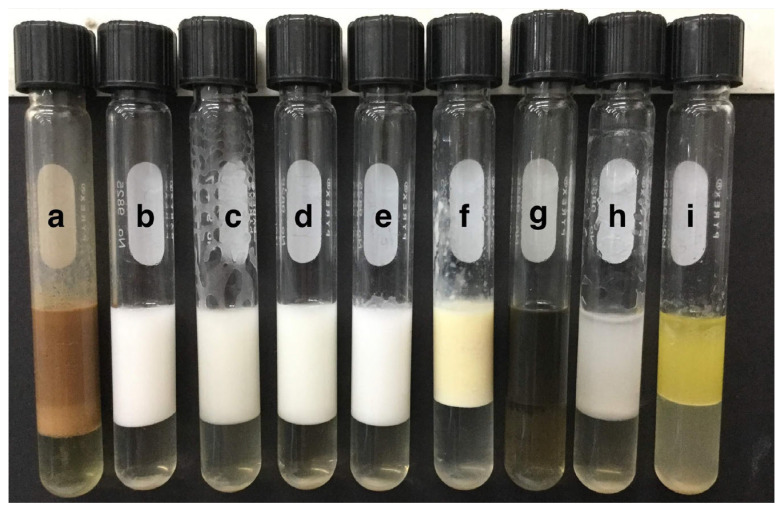
Emulsifying index obtained with different hydrocarbons using CL_84_ produced by *Bacillus subtilis* subsp. *spizizenii* MC6B-22. (**a**) Burnt motor oil, (**b**) Toluene, (**c**) Xylene, (**d**) Diesel, (**e**) *n*-Hexadecane, (**f**) Motor oil, (**g**) Crude oil (Petroleum), (**h**) *n*-Hexane, (**i**) Olive oil.

The CL_84_ formed stable emulsions with burnt motor oil, motor oil, *n*-hexadecane, xylene, toluene, *n*-hexane and crude oil (Figure 6 and Table 6). The gas oil was the only hydrocarbon not efficiently emulsified, whereas burnt motor oil and crude oil were the best emulsified substrates.

### 2.7. Stability of the Emulsifying Activity of CL84

The emulsifying stability of the purified fraction CL_84_ was evaluated using the substrate *n*-hexadecane under different temperatures, salinity and pH conditions. Temperature from 0 to 120 °C had no significant effect on EI_24_, emulsification even at 100 and 120 °C was slightly higher than at room temperature; only when CL_84_ was subjected to freezing conditions the emulsification decreased notably from 57.79 to 37.2% (Table 7). Regarding salinity, no changes were observed in the emulsification from 2 to 12% salinity. No significant effect was observed on the activity at different pH values. 

## 3. Discussion

### 3.1. Physiology, Biochemistry and Identification of Bacillus subtilis MC6B-22

According to Bergey’s manual of determinative bacteriology [27], the strain MC6B-22 was closely related to the *Bacillus* group, which consists of *B*. *mojavensis* and *B. subtilis* subsp. *spizizenii* NRRL B-23049 (Table 2), [44,45]. Generally, these taxa are closely related and indistinguishable from each other by their morphological, physiological and biochemical characteristics [46]. Previous phylogenetic analysis of *Bacillus* sp. MC6B-22 strain suggested it was a close relative of *B. mojavensis* and *B. subtilis* [47]. The phylogenetic trees based on the 16s and *gyrB* gene markers and genome sequence analyses suggest that MC6B-22 is closely related to *B. subtilis* subsp. *spizizenii*. This result was supported by genome sequence analysis compared with other species of the genus *Bacillus* [45,46,47,48,49,50].

### 3.2. Genetic Prospection of Lipopeptides

The biochemistry test suggested that MC6B-22 produces mycosubtilin and probably iturin. Mycosubtilin presence was confirmed by amplicon Sanger sequencing, which suggested that the MC6B-22 strain genome codifies for at least two lipopeptides (surfactin and mycosubtilin). The whole genome analysis suggests that MC6B-22 has other gene clusters involved in lipopeptides synthesis, such as surfactin, bacillaene, bacillibactin and bacilysin with known antibiotic activity, supporting the biochemical activity observed. Additionally, we detected other genome regions involved in the synthesizing of secondary metabolites that might have the biotechnological potential for plant pathogen control. For example, two regions with similarities to genes involved in terpene synthesis were identified. Recent work suggests that bacterial terpenes are a potential source of natural products with antimicrobial activity and are involved in microbe-host communication [30,51]. Interestingly, MC6B-22 has the gene cluster involved in rhizocticin A, which could explain the antifungal activity detected.

The chromatographic results suggest that the MC6B-22 strain also produces iturin, but the genome sequence did not confirm this. The iturin derivatives synthesis is performed by a hybrid complex that includes polyketide synthase and non-ribosomal peptide synthetase (PKSs/NRPSs) modules [52,53]. Indeed, mycosubtilin is a lipopeptide belonging to the iturin family [54]. Iturins are a group of antifungal cyclic lipopeptides produced commonly by the genus *Bacillus* [55]. The iturin group comprises iturin A–E, bacillomycin D-F, L and mycosubtilin [56]. All of them are cyclic peptides with seven α-amino acids (A1–A7) and one unique β-amino fatty acid (βAA). It is known that mycosubtilin and iturin only differ in the last two amino acids, which are inverted [52,57], and it is reported that nucleic sequences of synthetases from this lipopeptides family are on average, 76% similar [58]; our genomic sequence analysis showed only 44% of the mycosubtilin genes cluster are like similar to reported iturin gene clusters. 

Most of the *B. subtilis* subsp. *spizizenii* strains have been isolated from soil or have been associated with rhizospheric samples. Only gtP20 (recently reclassified as *B. subtilis* subsp. *inaquosorum*) [49,50] and DK1-SA11 [59] strains were isolated from marine samples. Nonetheless, their lipopeptide production has not been analyzed nor characterized. Therefore, this study is the first report of lipopeptides characterization and evaluation as antifungal agents from a marine *B. subtilis* subsp. *spizizenii* strain. Our results suggest that the sequence in the MC6B-22 strain has specific differences compared with other *B. subtilis* strains, probably due to its marine origin. As was proposed before, finding four LPs families in a *Bacillus* strain is unusual; those used as biocontrol agents usually have two or three families [60]. Our genomic analysis of the MC6B-22 marine strain suggests it produces other lipopeptides, which could mean a more complex LPs core for a *Bacillus* species.

### 3.3. Growth Kinetic and Production of Crude Lipopeptide from Bacillus subtilis MC6B-22 with Antifungal, Biosurfactant and Bioemulsifier Activities

The antifungal, biosurfactant and bioemulsifier activities produced during the growth of *B*. *subtilis* MC6B-22 were detected from 24 h of culture to the end of the stationary phase. These activities increased until reaching maximum activity at the death phase (84 h), when the highest number of endospores was also found. This indicates that the production of active biosurfactants is related to *Bacillus* sporulation. In general, lipopeptide production is induced when the cells have exhausted essential nutrients. For example, surfactin production is induced in actively growing cells during the transition from exponential to stationary phase, whiles fengycin synthesis is related to the early stationary phase and iturins only accumulate in the later stationary phase [52].

### 3.4. Partial Purification of Crude Lipopeptide Fraction

The crude extract of the biosurfactant lipopeptides prepurified by bioautography in TLC (PL_84_) coincided with the iturin and mycosubtilin standards, the zone of hemolysis and halo of inhibition of mycelial growth of *C. gloeosporioides*, demonstrating its antifungal activity. That fraction coincided with iturin and mycosubtilin standards by reverse-phase HPLC and MALDI-TOF mass spectrometry analysis. In nature, iturin A is produced as a mixture of up to eight isomers named iturin A_2_-A_8_ according to their types of βAA. The molecular masses of the isomers ranged from 1029 to 1084 [39]. The mycosubtilin and iturin A have almost the same structure, except that D-Ser_6_ and L-Ans_7_ residues in mycosubtilin are inverted to D-Ans_6_ and L-Ser_7_ in iturin [61].

### 3.5. Antifungal and Bioemulsifier Activities

The antifungal activity of crude lipopeptides of *Bacillus subtilis* MC6B-22 strain showed inhibition against all phytopathogenic fungi assayed, with a broad-spectrum antifungal effect, where the MIC values (in the order of μg/mL) suggest a good antifungal activity despite not being purified lipopeptides. Toral et al. [62] have shown that the butanolic extract of *Bacillus* sp. has a MIC and minimum fungicidal concentrations (MFC) of 8 mg/mL against *Botrytis cinerea.* Our present study demonstrates the potential of the lipopeptides produced by *Bacillus subtilis* against species of *Colletotrichum* and *Fusarium*, which were classified among the ten fungi most important phytopathogens worldwide [63]. For *Pestalotiopsis maculans* the effect was of fungistatic type; in the literature, it has been reported that this four-celled melanin fungus requires a high concentration of various organic and inorganic for showing fungicidal activity [64].

The broad inhibitory activity observed may be associated with the action of mycosubtilin, a class of pore-forming lipopeptides that is well recognized for its antifungal activity against a wide variety of pathogenic yeasts and fungi, but whose antibacterial activity is restricted to a few bacteria species. The lipopeptides, iturin and mycosubtilin, have been assigned as key factors in antagonism for *B*. *subtilis* [52]. However, the MC6B-22 strain has an interesting antimicrobial arsenal, which was confirmed by the lipopeptides genes clusters detected by the genome sequence analysis (mycosubtilin, surfactin, bacillaene, bacillibactin) and other interesting secondary metabolites such as bacilysin, subtilin, terpens, rhizocticin A, among others widely recognized for their antimicrobial activity.

Interestingly, the antifungal activity was evaluated against a strain type (*Colletotrichum gloeosporioides* ATCC 42374) and native phytopathogen fungi isolated from mango, papaya, guanabana, banana, rambutan and cocoa. The results support that *B. subtilis* subsp. *spizizenii* MC6B-22 strain is a strong candidate to be used as a biological control against diseases of agricultural interest.

On the other hand, the CL_84_ ability to emulsify different hydrocarbons using a wide range of pure and mixed substrates was demonstrated. Gudiña et al. [65] obtained similar results to gas oil, burnt motor oil and crude oil. Thanks to its broad-spectrum capacity to emulsify different substrates, the biosurfactant lipopeptide could be used in bioremediation. Not all biosurfactants have emulsifying activity, so CL_84_ has the duality of reducing surface tension and emulsifying, which is of interest in the bioremediation area.

The purified fraction of CL_84_ showed attractive emulsifying stability under the different temperatures, salinity and pH conditions assayed. It was noticeable that at pH 8 to 9, the emulsification was slightly higher than at pH 7; probably because at this pH the lipopeptides are in their ionized form. Therefore, they are more stable for the formation of emulsions [24]. The stability shown is favorable for bioremediation processes since the contaminated sites present extreme conditions such as high salinity and temperature during the hydrocarbon extraction processes or contaminated soil that is generally very acidic due to the disturbance by pollutants [13,66]. It was interesting to observe that the antifungal and hemolytic activity also remained stable under different temperatures, confirming that MC6B-22 lipopeptides are stable and highlighting their potential utility under various extreme conditions.

## 4. Materials and Methods

### 4.1. Morphology, Physiological and Biochemical of MC6B-22 Strain

The MC6B-22 strain was isolated from a marine biofilm and kept at deep-freezing condition (−80 °C) in tryptic soy broth (Difco Inc., Detroit, MI, USA) with 2.5% glycerol (*v*/*v*). This strain was evaluated for its physiological, morphological and metabolic characteristics. The pH was adjusted to 5.6 and the incubation temperature was 30 °C, except in growth tests at various temperatures. Growth at 50 °C was tested in the presence of 2, 5, 6.5 y 10% NaCl. Biochemical tests were determined using dextrose agar, hydrolysis of casein, starch, urea, tween 80, egg yolk lecithin, degradation of carboxymethyl cellulose, utilization of citrate, fermentation of glucose, mannitol and glycerol. The tests of Gram staining, mobility, Voges–Proskauer reaction, catalase and anaerobic growth were realized [27].

### 4.2. Phylogenetic Analysis of MC6B-22 and Genetic Characterization of Lipopeptides

Strain identification was based on 16s rDNA and *gyr*B molecular markers sequencing and analysis as described below. Genomic DNA from the MC6B-22 strain was isolated as described in Wilson (1997) [67].

The oligonucleotides used in the PCR amplifications are listed in Table S1. Briefly, the 20 μL reaction contained 50–100 ng/μL of DNA, 1 U of *Taq* DNA Polymerase (Invitrogen, Carlsbad, CA, USA), 0.2 mM of each deoxynucleoside triphosphate, 0.2 mM of an appropriate primer pair, 1X PCR buffer and 1.5 mM MgCl_2_ (except for fengycin reaction which included 1.0 mM MgCl_2_). Cycles (30 for lipopeptides and 16s and 32 for *gyr*B) are detailed in Table S1. All the reactions had a final extension of 72 °C for 10 min. The expected PCR products were purified with Quantum Prep PCR Kleen Spin columns (Bio-Rad, Hercules, CA, USA) and sequenced by the Sanger method (Macrogen sequencing service, Seoul, Korea). PCR products with multiple bands observed (i.e., plipastatin) were cloned using the CloneJet kit (Thermo Scientific, Waltham, MA, USA), transformed in *Escherichia coli* DH5-α and plasmid purified by QIAprep spin miniprep kit (Qiagen) before sequencing.

Amplicons were sequenced from both directions. They were analyzed with a BLAST search from NCBI [68] and KEGG [69] databases. Phylogenetic trees for 16s rDNA and *gyr*B were done with sequences from the SILVA rRNA database project (http://www.arb-silva.de/, accessed on 2 February 2023) [70]. The 16s phylogenetic analysis involved 30 sequences and 1268 positions in the final dataset. The evolutionary history was inferred using the Maximum Likelihood (ML) method based on the Kimura 2-parameter model + Gamma distribution ([+G], parameter = 0.1361) + Invariable sites [+I], 62.4783% [71]. The bootstrap consensus tree was inferred from 1000 replicates, using the Maximum Composite Likelihood (MCL) approach. For *gyr*B, the evolutionary history was inferred using the ML method based on the Tamura–Nei model + G, parameter = 0.9604 [72]. This analysis had 15 sequences and a total of 1014 positions. Using the MCL approach, the bootstrap consensus tree was inferred from 1000 replicates. Evolutionary analyses were conducted in MEGA6 [73].

### 4.3. Genome Sequencing and Analysis

To identify genomic regions involved in lipopeptide synthesis, we sequenced and analyzed the MC6B-22 genome. High molecular weight and high-quality genomic DNA were extracted and purified with the following protocol.

A colony from solid Luria–Bertani (LB) medium was taken and inoculated in a tube with 5 mL of LB broth at pH 8 supplemented with 1% Glucose. These were incubated at 36 °C with 150 rpm shaking for 48 h. Growth cells were harvested by centrifuging at 6000 rpm for 5 min to remove the culture medium. To the bacterial pellet, 180 μL of ATL lysis buffer from the DNeasy Blood and Tissue Kit (Qiagen, Hilden, Germany) was added, along with 5 μL of Proteinase K (20 mg/mL) and passed three times through a 31 G × 8 mm insulin needle (BD Ultrafine 0.3 mL). Subsequently, it was incubated at 65 °C for 5 min and 200 μL of AL buffer was added, continuing the incubation with the same conditions for another 10 min. The rest of the manufacturer’s protocol was followed and the DNA was eluted in 60 μL of elution buffer. We took 2 μL for electrophoresis in 0.8% agarose gel and visualized it with UV light. Another 2 μL were used for quantification in the NanoDROP Lite equipment (Thermo Scientific). 

The MC6B-22 whole genome was sequenced on the PacBio Sequel II platform with an 8 M SMRT Cell run (CCS/HiFi mode, 30 h movie, as part of 18× multiplex sequencing run) and assembled with the software Canu v2.2 [74] at Maryland Genomics.

The assembled and circularized genome was annotated with the RAST database to identify the number of coding sequences and RNAs. Benchmarking Universal Single-Copy Orthologs were searched with BUSCO 3.0.2 [75] as implemented in QUAST v. 5.2 [76], to evaluate assembly completeness regarding sequenced genes. The 16s rRNA gene regions were extracted to perform a BLAST search to confirm strain identity. We uploaded the genome sequence to the antiSMASH 6.0 database [77], to identify secondary metabolite biosynthesis gene clusters using the relaxed option that detects well-defined clusters containing all required parts and partial clusters missing one or more functional parts. 

### 4.4. Growth Kinetic and Production of Compounds with Antifungal, Hemolytic, Biosurfactant and Bioemulsifier Activity

The Luria–Bertani broth (Sigma Aldrich Co, St. Louis, MO, USA) with added sea salt 17.5 g/L (LBMs) and pH adjusted to 7.0 was used to produce biosurfactants. The experiment was carried out in baffled Erlenmeyer flasks of 250 mL with 90 mL of medium and were inoculated with 10 mL of overnight cultures adjusted to an optical density (OD_520 nm_) of 0.090, which corresponds to 1.5 × 10^8^ colony-forming units (CFU). The biomass was monitored at OD_520 nm_ and CFU/mL at each 12 h interval up to 132 h. The biosurfactant production was evaluated simultaneously after acid precipitation (see details below). All experiments were performed in triplicate and the results were averaged ± standard deviation.

#### 4.4.1. Extraction of Crude Lipopeptide

Crude lipopeptides were obtained each time (h) using the acid precipitation. Briefly, the cell-free supernatants (CFS) were obtained by centrifuging at 10,000 rpm at 4 °C for 20 min, followed by membrane filtration of 0.45 μm. The CFS were acidified by adding 6N HCl to achieve a final pH of 2.0, left to precipitate at 4 °C overnight and recovered by centrifugation at 6000 rpm for 45 min at 4 °C, to re-dissolve in alkaline water (pH 8.0). The solution was concentrated by lyophilization and was designated crude lipopeptides (CL) fraction. The weight of the CL fractions was determined and used to calculate the lipopeptides yield (weight of CL per dry weight of cells).

#### 4.4.2. Antifungal Activity

The antifungal activity was evaluated by the agar diffusion method of the CL obtained at different times using the phytopathogenic fungus *Colletotrichum gloeosporioides* (ATCC 42374). From a 7-day culture, the inoculum was prepared by filtering the conidia with sterile gauze in solution with NaCl at 0.85% and Tween 20 (0.025% *v*/*v*) and adjusted to 1.6 × 10^5^ conidia/mL with a hemocytometer. One mL of conidia suspension was deposited on the plates with Potato Dextrose Agar (PDA Difco Laboratories, Detroit, MI, USA), spreading the inoculum evenly. Wells of 7 mm diameter were drilled and 70 μL of CLs (10 mg/mL) were placed for each evaluated time. The plates were incubated at 28 °C for 72 h. The assay was performed in triplicate and checked daily for antifungal activity. The inhibition halos were expressed in mm [64], and Daconil^®^ (2 mg/mL), was used as a control positive.

#### 4.4.3. Hemolytic Activity

The hemolytic activity of the CL obtained at different times was evaluated since it has been documented that hemolysis can suggest the presence of lipopeptides [78]. In blood agar plates freshly prepared with 5% (*v*/*v*) sheep blood, 7 mm diameter wells were punched and filled with 70 μL of the CLs (10 mg/mL). The plates were incubated at 25 °C for 24 h. All tests were performed in triplicate and hemolytic activity visualized by developing a clear zone (halo) around the well was recorded in the inhibition zone diameter values (mm).

#### 4.4.4. Drop-Collapsing Test

Along with the hemolytic activity, the CLs was examined using the drop-collapsing test because it has been shown that lipopeptides do not necessarily respond positively to both tests [79]. This technique is an indirect method used to measure the biosurfactant capacity of a compound. This test was performed on a 96 microwell plate lid (Nunc, Roskilde, Denmark). Two microliters of mineral oil were placed and stabilized for 24 h and then 5 μL of CL suspended in distilled water (10 mg/mL) were added to the surface of the oil. The shape of the drop on the oil surface was inspected and measured after 1 min under the microscope. The CL fractions recovered at the different times that gave flat drops ≥4 mm were considered positive [79]. As a negative control, distilled water and sterilized media (LBMs) were used; the positive control was the commercial surfactants Triton^TM^ X-100. 

#### 4.4.5. Emulsifying Activity Determination

The emulsifying activity was determined in tubes by adding 2 mL of CL_84_ (10 mg/mL) and 2.0 mL of *n*-hexadecane; this was mixed vigorously in a vortex (3000 rpm) for 2 min, leaving it to stand in the dark for 24 h at room temperature [80]. The emulsification index was defined as the height of the emulsification divided by the total height, expressed as a percentage. The capacity of the stable bioemulsifier in different hydrophobic substrates was evaluated and the *n*-hexadecane was replaced in the emulsification assays by burnt motor oil, toluene, xylene, diesel, motor oil, crude oil (petroleum), *n*-hexane and olive oil. All emulsification indexes were performed in triplicate, as controls were used commercial surfactant anionic Sodium Dodecyl Sulphate (SDS) and surfactants no-anionic Triton^TM^ 100X and Tween 80.

### 4.5. TLC Bioautography Analysis of CL84

The highest yield of the CL fraction by *B. subtilis* var. *spezizenii* was obtained at 84 h of fermentation and referred as (CL_84_.) Therefore, fermentation was carried out at a volume of 2 L under the same conditions to recover higher amounts of CL for purification and characterization. The CL_84_ fraction was characterized following a modified version of the standard bioautographic TLC method. The CL_84_ fraction was extracted three times with methanol and 100 μL of the methanolic fraction (1 mg/mL) was applied on two preparative thin-layer chromatography (TLC) silica gel plates (Merck, 0.2 mm, 60 F25). The TLC plates were eluted with the solvent system butanol:methanol:water (3:2:1, *v*/*v*/*v*) and dried at room temperature for 15 min. 

Into the first TLC plate, saline (0.85%) and agar (1%) mixed with sheep blood (5%) were poured slowly under aseptic conditions. The agar layer was allowed to solidify and the plate was incubated at 25 °C for 24 h. The presence of areas of hemolysis in the plates was verified. For antifungal activity, molten soft PDA (1.0% agar) was poured onto a plate and inoculated with 1 × 10^5^ conidia/mL of *C. gloeosporioides* (ATCC 42374) on TLC. The plate was incubated at 28 °C and the mycelium’s growth and the inhibition zones were verified after 48 h. The bioautography was stained with a 2,3,5-triphenyl tetrazolium chloride (TTC, Sigma Aldrich) to observe fungal growth inhibition. Fungal growth reduces tetrazolium compounds to a deep red color from formazan derivatives. The zones of hemolysis and inhibition were used to calculate the retention factor (R_f_).

### 4.6. Partial Purification and Characterization of Crude Lipopeptide Fraction

A larger amount of lipopeptides was purified with 300 mg of CL_84_ on a new preparative TLC plate using the same solvent system. The R_f_ values matching hemolytic activity and lipopeptide were used to recover CL_84_ by scraping the TLC plate. The recovered silica gel was eluted with 80 mL of chloroform:methanol (2:1) overnight, filtered and concentrated by solvent evaporation on a rotary evaporator. The antifungal and hemolytic activities of this purified lipopeptide fraction (PL_84_) were confirmed by an additional bioautography TLC assay. At the same time, the chemical nature of active compounds was determined with other TLC plates revealed with 0.2% ninhydrin in absolute alcohol (followed by heating to 110 °C) to detect peptides and with 10% cupric sulfate in phosphoric acid for lipids.

#### 4.6.1. Reverse-Phase HPLC Analysis

The purified lipopeptide fraction (PL_84_) was characterized by reverse-phase high-pressure liquid chromatography (HPLC) with a 1260 infinity chromatograph (Agilent Technologies, Santa Clara, CA, USA) with a C18, 4-mm Zorbax, Agilent (100 × 4.6 mm) column. Samples (10 mL) of PL_84_ and mycosubtilin standard (Lipofabrik, Lesquin, France) were analyzed at a flow rate of 0.3 mL/min with an isocratic elution using 60% solvent A (water) and 40% solvent B (acetonitrile) at 20 °C. Peaks eluting from the column were detected by their absorbance at 220 nm [43].

#### 4.6.2. Mass Spectrometry

The molecular masses of the HPLC purified isoforms were determined by a 4800 MALDI-ToF/ToFTM analyzer (Applied Biosystems Inc., MDS SCIEX, CA, USA) equipped with a nitrogen UV laser (337 nm) operated at 10 Hz for the desorption and ionization of the molecules [81]. The matrix DHB (2,5-dihydroxybenzoic acid) (Sigma Aldrich Co, St. Louis, MO, USA) was used to co-crystallize the compound. The 10 mg/mL concentration of matrix stock was the samples volume and the matrix was mixed for 5 min on a vortex mixer. The well-mixed sample was spotted on a target plate, then dried and placed inside the sample cabinet of the instrument. The acceleration voltage of 20 kV was applied to accelerate the molecules. The molecules were separated according to their mass ions and were detected by a linear ion detector set at reflector mode. The molecular mass gate of 500 Da was provided to avoid the noise for better precision and the instrument was calibrated externally with a mass accuracy of 0.01%.

### 4.7. Isolation of Different Phytopathogens

The antifungal activity was evaluated against *Colletotrichum gloeosporioides* (ATCC 42374) and native strains as the causal agent of anthracnose, *C*. *gloeosporioides* in mango (*Mangifera indica* L.), *C*. *gloeosporioides* and *C*. *capsici* in papaya (*Carica papaya* L.) were isolated. In addition, other phytopathogenic strains were isolated from a range of tropical fruit hosts. *Fusarium solani* in Guanabana (*Annona muricata* Linn), *F. nivale*, *Ascochyta* sp.; and *Curvularia clavata* in banana (*Musa acuminata* Colla), *Pestalotiopsis maculans* from rambutan fruits (*Nephelium lappaceum* L.) and *Moniliophthora roreri* was isolated from cocoa (*Theobroma lappaceum* L.). All these fungi cause severe diseases in tropical fruits leading to high economic losses for farmers. Koch’s postulates confirmed fungal pathogenicity. The strains isolated were identified according to macroscopic and microscopic characteristics such as mycelia, conidiogenesis, arrangement of conidia, among others using taxonomic keys and consulting specialized references. All isolates were conserved on PDA in sterile water at room temperature. 

### 4.8. Fungicidal and Fungistatic Activity

The activity of the fraction (CL_84_) was evaluated by the well diffusion assay against the different pathogens as mentioned above (4.4.2) and purified lipopeptide fraction (PL_84_) with the microdilution assay to determine the minimum inhibitory concentrations (MIC) and minimum fungicidal concentrations (MFC) according to M38-A2 CLSI [82]. In 96-well microplates (Nunc F96), 100 μL of RPMI 1640 medium (0.165 M MOPS, pH 7.0, with L-glutamine, without NaHCO_3_ medium) containing PL_84_ in two-fold serial dilutions (100–0.390 μg/mL) and 100 μL of a suspension of 1 × 10^4^ conidia/mL were deposited. The commercial fungicide Daconil^®^ 100 μg/mL and uninoculated medium were used as positive and negative controls, respectively. The plates were incubated for 48 h at 37 °C. The concentrations in which fungal growth is inhibited (MIC) were determined visually using optical microscopy (Carl Zeiss, Oberkochen, Germany). To determine MFC, 10 μL of each well was taken and inoculated to a new PDA media to incubate for 72 h at 28 °C; if there was no mycelial growth, it was determined as a fungicidal effect. The PL_84_ was considered fungicidal when the MFC/MIC ratio was ≤4 and fungistatic when the MFC/MIC ratio was >4 [83,84].

### 4.9. Effect of Salinity, pH and Temperature on Bioemulsifier Activity

Stability studies of bioemulsifier activity were performed using CL_84_ prepared in distilled water (pH 8) at a concentration of 1 g/L. CL_84_ solutions were supplemented with different NaCl concentrations (from 20, 40, 80, 100 and 120 g/L) to test the effect of salinity. Stability at different temperatures was evaluated by incubating the CL_84_ solutions at 0, 5, 70 and 100 °C for 1 h and at 120 °C for 20 min; the samples were then cooled to room temperature and the emulsification indexes were measured. The pH stability was studied by adjusting the CL_84_ solutions to different pH values (2–12) using HCl or NaOH solutions and then the emulsifying activity was measured as previously described [65,85]. All the experiments were carried out in triplicate.

## 5. Conclusions

*Bacillus subtilis* subsp. *spizizenii* MC6B-22 produces secondary metabolites with broad-spectrum biosurfactant, bioemulsifier and antifungal activities against various phytopathogens, strongly impacting tropical fruits of great global demand, especially against *Colletotrichum gloeosporioides* and *Moniliophthora roreri*, with a mode of action fungicidal at low concentrations (25 μg/mL). The production of lipopeptides with acceptable biological activity was recorded from 48 to 96 h, coinciding with the start of the stationary and death phases. However, at 84 h, the production of lipopeptides with the highest biosurfactant, bioemulsifying, hemolytic and antifungal activity was found; at this time, the formation of endospores was observed. The identification of lipopeptides such as mycosubtilin was confirmed by HPLC and MALDI-TOF. Genomic sequences confirmed the identities of mycosubtilin, surfactin, bacillaene and bacillibactin, although gene clusters for iturin were not identified. Furthermore, sequences for bacilysin, subtilin, terpenes and rhizocticin A, among other genes related to antimicrobial activity, were detected. In addition, the antifungal, biosurfactant and bioemulsifier activities remain stable when subjected to different pH, temperatures and salinity. Our results show the potential of the MC6B-22 strain for agricultural and bioremediation fields; however, further studies at the microcosm and field level are necessary to determine its effectiveness in situ.

## Figures and Tables

**Table 1:** Morphological, physiological and biochemical characteristics of *B*. *subtilis* subsp. *spizizenii* MC6B-22.

Characteristics			
Gram reaction	+	**Degradation of**	
Spore shape	Oval	Carboxymethyl cellulose	−
Growth microaerophilic	+	**Use of**	
Catalase	+	Citrate	+
Oxidase	+	**Growth at**	
Motile	+	pH 5.6	+
Nitrate reduction	+	50 °C	+
**Production of**		**Growth in presence of**	
Indole	−	LBM	+
H_2_S	−	2% NaCl	+
pH in Vosges-Proskauer	+	5% NaCl	+
**Hydrolysis of**		6.5% NaCl	+
Casein	+	10% NaCl	+
Starch	+	**Fermentation of**	
Urea	+	Mannitol	−
Tween 80	−	Glycerol	−
Egg yolk lecithin	−	Glucose	+

**Table 2:** *B. subtilis* subsp. *spizizenii* MC6B-22 genome characteristics compared with the top three hits.

Matched Strain	Score Range	Overlap	e-Value	Identity Range	Acc. Number	16S RNA Length	16S RNA Copy Number
*Bacillus subtilis* strain FDAARGOS_606	2874	100%	0	100%	CP041015.1	1556	10
*Bacillus subtilis* subsp. *spizizenii* str. W23	2868–2874	100%	0	99.94–100%	CP002183.1	1556	10
*Bacillus intestinalis* strain T30	2868–2874	100%	0	99.94–100%	CP011051.1	1556	10

**Table 3:** antiSMASH detected regions of secondary metabolite biosynthesis gene clusters in the MC6B-22 genome.

N	Type	Location	Most Similar Known Cluster (Similarity ^1^)	Most Similar Known Cluster Type	Activity
1	TransAT-PKS, PKS-like, T3PKS, NRPS	430,447–535,671	Bacillaene (100%)	Polyketide + NRP	Polyene antibiotic that is active against a wide range of bacteria [28].
2	NRPS, betalactone, transAT-PKS	602,362–678,373	Mycosubtilin (100%)	NRP + Polyketide	Polypeptide with antifungal and hemolytic activities, belonging to the iturin lipopeptide family [29].
3	Terpene	795,235–816,705			Diverse functions: infochemicals for inter and intraspecies communication, response to temperature, oxidative and osmotic stress, antimicrobial, anti-oxidative, anti-inflammatory and anti-cancer [30].
4	T3PKS	864,155–905,252	1-carbapen-2-em-3-carboxylic acid (16%)	Other	Antibiotic with activity against Gram-positive and Gram-negative bacteria [31].
5	NRP-metallophore, NRPS, RiPP-like	1,759,322–1,811,439	Bacillibactin (100%)	NRP	Catecholate siderophore capable of binding and solubilizing iron. It chelates Fe^3+^ from the environment, which is later transported into the bacterial cytoplasm. Some Bacillibactins have shown bactericidal activity against multidrug-resistant strains [32].
6	Lanthipeptide-class-i	1,962,423–1,988,648	Subtilin (100%)	RiPP:Lanthipeptide	Lantibiotic originally produced by *Bacillus subtilis* ATCC6633. It shows antimicrobial activity against Gram-positive bacteria [33].
7	CDPS	2,094,187–2,114,933	Pulcherriminic acid (100%)	Other	Cyclic dipeptide that chelates Fe^3+^. This property allows competition for environmental iron ions to achieve bacteriostatic effects [34].
8	Sactipeptide, other	2,351,558–2,413,975	Bacilysin (100%)	Other	Induction of bacteria and fungi cell wall lysis [35].
9	Phosphonate	2,951,523–2,971,909	Rhizocticin A (100%)	Other	Hydrophilic phosphono-oligopeptide with antifungal activity [36].
10	NRPS	3,098,092–3,163,483	Surfactin (86%)	NRP: Lipopeptide	Antibacterial, antifungal and antiviral agent; surfactant, antineoplastic and a platelet aggregation inhibitor; cyclodepsipeptide and a macrocyclic lactone [37].
11	Terpene	3,867,459–3,888,265	No match	No match	Many putative terpene synthase genes have been discovered in bacteria [38].

N, region number; ^1^, Percentage of genes with similarity to known cluster.

**Table 4:** Growth kinetic and antifungal, hemolytic, biosurfactant and bioemulsifiying activities monitored for 132 h in LBMs of *Bacillus subtilis* subsp. *spizizenii* MC6B-22. The antifungal activity shown was against *C. gloeosporioides* ATCC 42374.

Time (h)	OD_520 nm_	Vegetative Cells (CFU/mL)	Spores (CFU/mL)	pH	CL (mg/L)	Antifungal (mm)	Hemolyticg (mm)	Biosurfactan ^1^ (mm)	Emulsifying EI_24_ (%)
12	1.94 ± 0.20	2.33 × 10^8^	4.86 × 10^5^	7.51 ± 0.04	289 ± 80	(–)	(–)	4.0	(–)
24	3.13 ± 0.23	5.36 × 10^8^	6.00 × 10^5^	7.90 ± 0.04	239 ± 70	12	(–)	4.0	5
36	3.35 ± 0.31	6.54 × 10^8^	5.83 × 10^6^	8.36 ± 0.01	479 ± 10	19	9	4.5	49.52
48	5.53 ± 0.20	3.21 × 10^9^	3.60 × 10^7^	8.23 ± 0.03	359 ± 11	24	14	5.6	58.13
60	6.96 ± 0.58	3.50 × 10^11^	1.69 × 10^7^	8.22 ± 0.00	366 ± 02	25	14	5.8	58.71
72	5.86 ± 0.42	1.41 × 10^11^	2.69 × 10^9^	8.57 ± 0.02	435 ± 03	22	13	5.0	59.01
**84**	**5.04 ± 0.15**	**7.72 × 10^10^**	**3.82 × 10^10^**	**8.49 ± 0.01**	**556 ± 79**	**27**	**15**	**6.0**	**59.03**
96	5.85 ± 0.41	5.14 × 10^10^	1.96 × 10^10^	8.71 ± 0.02	481 ± 05	24	13	5.0	56.81
108	4.09 ± 0.21	2.74 × 10^10^	1.73 × 10^12^	8.58 ± 0.04	537 ± 75	23	13	4.3	(–)
120	3.88 ± 0.16	1.14 × 10^10^	1.96 × 10^13^	8.73 ± 0.02	712 ± 15	23	13	4.0	(–)
132	3.08 ± 0.19	2.16 × 10^9^	3.71 × 10^13^	8.82 ± 0.03	502 ± 01	(–)	(–)	(–)	(–)

^1^ Drop collapsing test results. (–), not activity.

**Table 5:** Broad-spectrum antifungal activity of crude lipopeptides (CL_84_) of *Bacillus subtilis* subsp. *spizizenii* MC6B-22.

Fungal Phytopathogen	Reported Disease	Antifungal Activity				
		Inhibition Diameter (mm)	MIC μg/mL	MFC μg/mL	Relation MFC/MIC	Action Mode
*Ascochyta* sp.	Leaf blotch	24	12.5	100	1	Fungicide
*Colletotrichum acutatum*	Anthracnose	20	25	25	1	Fungicide
Colletotrichum gloeosporioides *	Anthracnose	27	25	100	4	Fungicide
*Colletotrichum gloeosporioides*	Anthracnose	28	25	100	4	Fungicide
*Colletotrichum capsici*	Anthracnose	24	25	50	1	Fungicide
*Curvularia clavata*	Leaf spot	25	400	400	4	Fungicide
*Fusarium nivale*	Fusariosis	17	100	400	1	Fungicide
*Fusarium solani*	Fusariosis	15.5	100	400	4	Fungicide
*Moniliophthora roreri*	Frosty pod rot	34	100	400	4	Fungicide
*Pestalotiopsis maculans*	Leaf spot	26	50	400	16	Fungistatic

* Strain ATCC 42374.

**Table 6:** Emulsifying activity EI_24_ (%) of CL_84_ produces by *Bacillus subtilis* subsp. *spizizenii* MC6B-22 in presence of different hydrophobic substrates.

	Hydrocarbons	CL_84 (%)_	SDS	Triton 100-X
Alkanes	*n*-Hexadecane	57.79	58.33	56.48
	*n*-Hexane	54.90	58.33	57.84
	Crude oil (petroleum)	50.98	47.56	47.56
	Olive Oil	44.69	48.80	59.24
	Diesel (gas oil)	59.00	65.21	57.50
	Burnt motor Oil	66.67	49.41	75.93
	Motor Oil	56.76	83.06	49.39
Aromatics	Toluene	61.90	60.71	62.20
	Xylene	59.95	61.18	61.63

**Table 7:** Stability of the emulsifying activity of CL_84_ under different pH, salinity and temperature conditions.

pH	EI_24_ (%)	Salinity (NaCl %)	EI_24_ (%)
2	36.2	2	57.2
3	48.9	4	59.0
4	52.3	8	57.9
5	53.9	10	57.3
6	53.8	12	56.40
7	59.3	**Temperature (°C)**	**EI_24_ (%)**
8	65.0	0	37.2
9	60.0	5	55.3
10	51.6	70	56.2
11	48.3	100	60.8
12	56.6	120	60.4

## Data Availability

On inquiry, the data presented in this study is available from the authors.

## Supplementary Materials

The following supporting information can be downloaded at: https://www.mdpi.com/article/10.3390/plants12061374/s1, Figure S1: Comparison of *B. subtilis* subsp. *spizizenii* strain MC6B-22 showing percentage of genes matching known gene clusters for secondary metabolite biosynthesis. BLAST analysis and diagram was produced with antiSMASH 6.0 (https://antismash.secondarymetabolites.org; accessed on 18 January 2023); Table S1: Primer and cycles for PCR amplification and lipopeptides biosynthesis genes in MC6B-22. Refs. [86,87,88,89,90] are cited in Supplementary Materials.
