# Supplementary material for: Broad-Spectrum Antifungal, Biosurfactants and Bioemulsifier Activity of Bacillus subtilis subsp. spizizenii—A Potential Biocontrol and Bioremediation Agent in Agriculture
Source: Plants (Basel). 2023 Mar 20;12(6):1374. doi: 10.3390/plants12061374 (PMC10056679; doi:10.3390/plants12061374)
Supplement: Supplementary file 1 [file plants-12-01374-s001.zip › plants-2244865-supplementary.pdf]

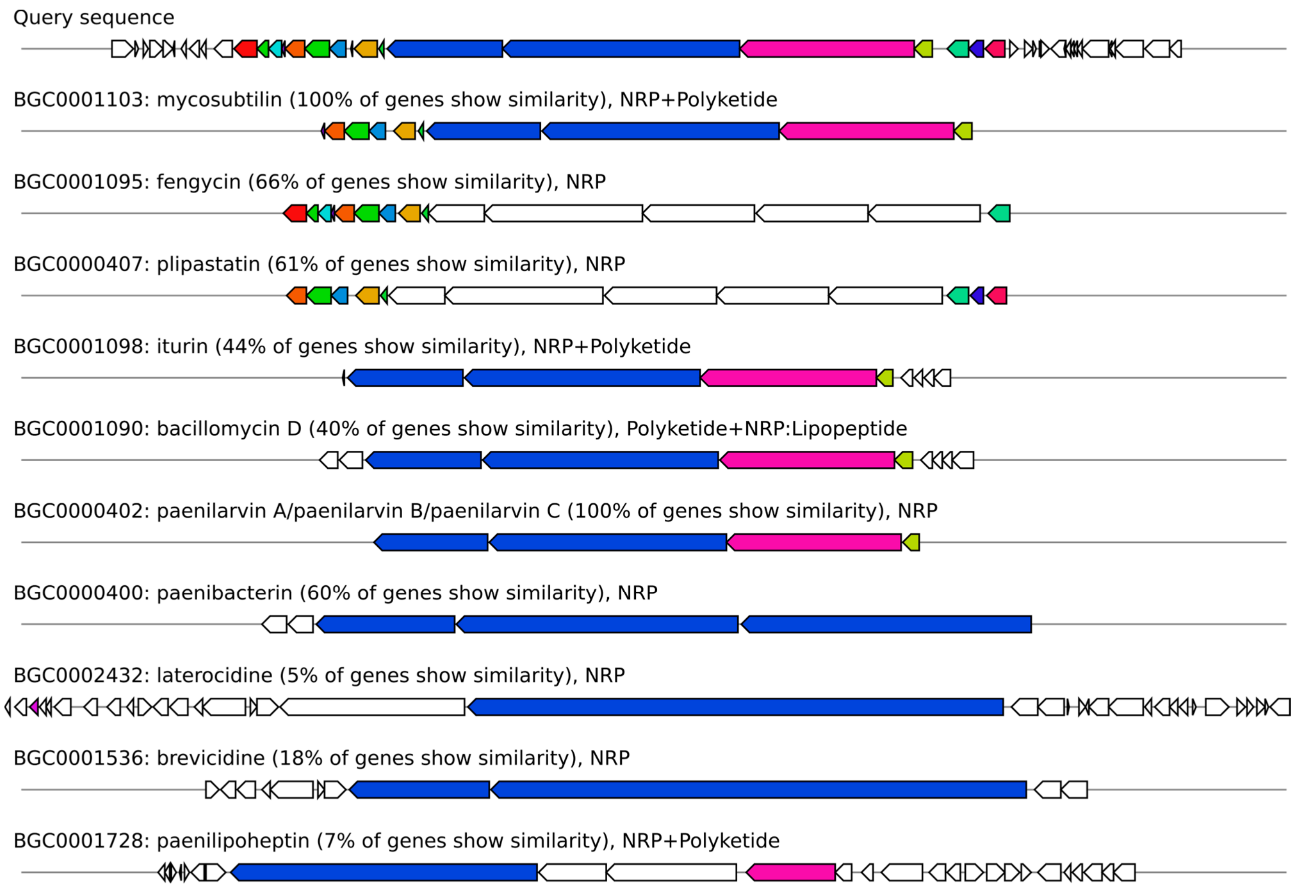

Figure S1. Comparison of *B. subtilis* subsp. *spizizenii strain* MC6B-22 showing percentage of genes matching known gene clusters for secondary metabolite biosynthesis. BLAST analysis and diagram was produced with antiSMASH 6.0 (https://antismash.secondarymetabolites.org).

Table S1. Primer and cycles for PCR amplification and lipopeptides biosynthesis genes in MC6B-22.

Family Lipopeptide	Temperature cycling used °C/seg	Primer name	Sequence 5′-3′	Product length[a]	Reference
Surfactins	94/60<br>48/30<br>72/45	As1-F	CGCGGMTACCGVATYGAGC	0.42	[58]
		Ts2-R	ATBCCTTTBTWDGAATGTCCGCC		
Mycosubtilins	94/60<br>48/30<br>72/45	Am1-F	CAKCARGTSAAAATYCGMGG	0.42	
		Tm1-R	CCDASATCAAARAADTTATC		
Fengycins	94/30<br>48/30<br>72/30	Af2-F	GAATAYMTCGGMCGTMTKGA	0.45	
		Tf1-R	GCTTTWADKGAATSBCCGCC		
Plipastatins	94 30<br>50/30<br>72/90	Ap1-F	AGMCAGCKSGCMASATCMCC	0.90	
		Tp1-R	GCKATWWTGAARRCCGGCGG		
Iturin	94/30<br>55/30<br>72/150	ITUP1-F	AGCTTAGGGAACAATTGTCATCGG GGCTTC	2.0	[86]
		ITUP2-R	TCAGATAGGCCGCCATATCGGAAT GATTCG		
Bacillomycin D	Various conditions were tested	BACC1F	GAAGGACACGGCAGAGAGTC	0.87	[87]
		BACC1R	CGCTGATGACTGTTCATGCT		
Kurstakins	94/60<br>40/30<br>72/45	Aks-F	TCHACWGGRAATCCAAAGGG	1.2	[88]
		Tks-R	CCACCDKTCAAAKAARKWATC		
		Tks-R	CCACCDKTCAAAKAARKWATC		
16s rDNA	95/30<br>53/30<br>72/60	fD1	AGAGTTTGATCCTGGCTCAG	1.5	[89]
		rD1	AAGGAGGTGATCCAGCC		
*gyr*B	94/30<br>60/30<br>72/120	gyrBBAU P2	GCGGAAGCGGCCNGSNATGTA	~1.5	[90]
		gyrBBND N1	CCGTCCACGTCGGCRTCNGYCAT		

[a] Kilo base pairs expected as reported at literature.

References

86. Tsuge, K.; Inoue, S.; Ano, T.; Itaya, M.; Shoda, M. Horizontal transfer of iturin A operon, itu, to *Bacillus subtilis* 168 and conversion into an iturin A producer. *Antimicrob. Agents Chemother.* **2005**, *49*, 4641–4648.
87. Ramarathnam, R.; Bo, S.; Chen, Y.; Fernando, W.G.D.; Xuewen, G.; de Kievit, T. Molecular and biochemical detection of fengycin and bacillomycin D-producing *Bacillus* spp.; antagonistic to fungal pathogens of canola and wheat. *Can. J. Microbiol.* **2007**, *53*, 901–911.
88. Abderrahmani, A.; Tapi, A.; Nateche, F.; Chollet, M.; Leclère, V.; Wathelet, B.; Hacene, H., Jacques, P. Bioinformatics and molecular approaches to detect NRPS genes involved in the biosynthesis of kurstakin from *Bacillus thuringiensis*. *Appl. Microbiol. Biotechnol.* **2011**, *92*, 571–581.
89. Weisburg, W.G.; Barns, S.M.; Pelletier, D.A.; Lane, D.J. 16s ribosomal DNA amplification for phylogenetic study. *J. Bacteriol.* 1999, *173*, 697–703.
90. Santos, S.R.; Ochman, H. Identification and phylogenetic sorting of bacterial lineages with universally conserved genes and proteins. *Environ. Microbiol.* **2014**, *6*, 754–759.
